# Evidence for Sex-Segregated Ocean Distributions of First-Winter Wandering Albatrosses at Crozet Islands

**DOI:** 10.1371/journal.pone.0086779

**Published:** 2014-02-27

**Authors:** Susanne Åkesson, Henri Weimerskirch

**Affiliations:** 1 Department of Biology, Lund University, Lund, Sweden; 2 Centre d'Etudes Biologiques de Chizé, Centre National de la Recherche Scientifique, Villiers en Bois, France; Institute of Ecology, Germany

## Abstract

The highly mobile wandering albatrosses (*Diomedea exulans*) are adapted to navigate the extreme environment of the Southern Ocean and return to isolated islands to breed. Each year they cover several hundreds of thousands of kilometers during travels across the sea. Little is known about the dispersal flights and migration of young albatrosses. We tracked, by satellite telemetry, the departure dispersal of 13 juvenile wandering albatrosses from the Crozet Islands and compared them with tracks of 7 unrelated adults during the interbreeding season. We used the satellite tracks to identify different behavioural steps of the inherited migration program used by juvenile wandering albatrosses during their first solo-migration. Our results show that the juvenile wandering albatrosses from Crozet Islands moved to sex-specific foraging zones of the ocean using at departures selectively the wind. The results suggest that the inherited migration program used by the juvenile wandering albatrosses encode several distinct steps, based on inherited preferred departure routes, differences in migration distance between sexes, and selective use of winds. During long transportation flights the albatrosses were influenced by winds and both adult and juveniles followed approximate loxodrome (rhumbline) routes coinciding with the foraging zone and the specific latitudes of their destination areas. During the long segments of transportation flights across open seas the juveniles selected routes at more northerly latitudes than adults.

## Introduction

Wandering albatrosses are avian masters of oceanic flight and famous for their long-distance migrations and foraging trips across open waters in one of the world's most extreme environments, the Southern Ocean around the Antarctic Continent [1]–[3]. Throughout their life-time the adult wandering albatrosses return every second year to their breeding sites located on isolated islands around the Antarctic Continent to reproduce. The sabbatical year between breeding attempts is spent in the open sea environment during which time the individual bird limits its movements to a restricted area of the ocean [3], [4]. The life of wandering albatrosses is characterised by their undisputed navigational abilities, enabling them to successfully locate isolated breeding islands during consecutive breeding attempts and when returning to the islands from foraging trips, but also to navigate during migrations covering thousands of kilometres across open seas, e.g. [5]. Despite these impressive migrations and navigation abilities, the first migration by young wandering albatrosses is not well understood, and it is not known by which cues the albatrosses navigate across open sea as well as to relocate the breeding islands, e.g. [4], [6]–[10].

Many songbirds migrate alone and rely on a genetic program to guide them across completely unknown terrain to their first winter destination [11]. The genetic migration program used by young birds has been proposed to be based on a simple “clock-and-compass” model (reviewed by e.g. [12], [13]), encoding distance and direction of migration set by the birds' internal time sense and the biological compasses relying on information from the Sun, stars and the geomagnetic field, e.g. [14]–[16]. As an alternative to this model it has been suggested that the first-year songbird migrant might navigate to a population-specific and pre-programmed geographic goal. According to this model the juvenile bird might even head for intermediate goals along the migration route (i.e. “moving-goal-area”) originally proposed for young songbirds by Rabøl [17]. The model was proposed as a mechanism to cope with unexpected displacements, for example, caused by orientation errors and uncorrected wind drift during migration flights to enable the terrestrial birds to stay on route during long-distance migrations. The “moving-goal-area” navigation model has rendered some support from ringing recovery analyses, e.g. [18] and from simulated magnetic displacements [19] for terrestrial songbirds, suggesting that long-distance migrating birds indeed head for intermediate areas of importance, for example before crossing large barriers to reach their final winter destinations. Songbirds have been shown to also react to simulated magnetic displacements by increasing their fat deposition before barrier-crossing [19], suggesting influence from external cues on their inherited physiological program. These intermediate areas used for refuelling on stop-over have been shown for a number of passerine migrants to be species-specific and very restricted in geographic range [18].

Whether young seabirds in general, and young albatrosses in particular, rely on a simple “clock-and-compass” mechanism, e.g. [12], [13] or navigate to population-specific destination areas or even to intermediate goals along the migration route [17] during their first solo-migration is not known. The advancement of tracking technologies to follow the migration of individual birds by satellite telemetry have enabled us to start to investigate these models explaining the navigation performances of young wandering albatrosses during their first migration. By analysing the routes followed by individual birds on natural migration we identified the different components of the migration program used by the young wandering albatrosses. We compared these movement patterns with those recorded for unrelated adults leaving the breeding sites at Crozet Islands after a breeding attempt and relate their movements to their first year destination areas of the ocean.

We wanted if possible to identify specific behavioural steps expressed during the first migration by the juvenile wandering albatrosses and investigate whether these steps would indicate that the young albatrosses rely on a simple genetic program encoding distance and direction to reach the migration goal [12], [13], or if their movements indicated inherited navigation skills to reach population-specific intermediate or terminal ocean areas. We investigated if the wandering albatrosses during the migration flights were following approximate orthodromic (i.e. great circle) or loxodromic (i.e. rhumbline) routes [20] to reach these distant areas at sea, from the initial location of the breeding island or from areas reached later during migration. Following an orthodromic route will lead to the birds along the shortest flight route between locations, while the loxodrome route is slightly longer, but simpler in terms of navigation, since it is a route following a constant geographic course.

We set up four hypotheses covering the alternative scenarios according to which we expected the young albatrosses to depart from the breeding island on dispersal flights to the wintering area at sea. As a null hypothesis for hypothesis 1–3 below, we expected the young albatrosses to leave the island in any direction and search for foraging areas randomly around the island, while for hypothesis 4 we expected that the initial departure path and ocean area used for foraging were not different from the adult wandering albatrosses. According to Hypothesis 1, we expected the young albatrosses to follow an orthodromic route leading from the site of birth to a restricted oceanic foraging zone. Hypothesis 2: the young albatrosses were expected to follow a loxodromic route from the breeding island to the ocean foraging zone. Hypothesis 3: the young albatrosses were expected to follow a combination of routes (as outlined in Hypothesis 1 and 2 above). Hypothesis 4: we expected the initial departure routes in juveniles to differ from those used by the adult albatrosses. The overall aim of this study was to investigate if movement patterns differed between different sex and age groups.

## Results

We tracked the migration of 13 juvenile wandering albatrosses lasting between 2 and 13 months (average 5.6 months) during their first departure from the breeding colony at Crozet Islands and 7 non-related adults (2 tracked by satellite telemetry and 5 with light loggers) [12], [13] lasting at least 4 months. These tracks cover the migration of individual albatrosses to an individually preferred ocean foraging zone (Table 1), and movements within the oceanic foraging zone where the birds spend the non-breeding period [3].

**Table 1:** Mean latitude and longitude of oceanic foraging zone for different age classes and sexes of wandering albatrosses breeding at Crozet Island as tracked by geolocators and satellite telemetry in different years.

Age	Sex	Indivudual	Year	Latitude (South)	Longitude (East)	Tracking method
Adult	Female	F1	1996	−34.989°	49.740°	Geolocator
Adult	Female	F3	1996	−43.753°	49.722°	Geolocator
Adult	Female	72448	2007	−41.421°	29.804°	Satellite telemetry
Adult	Male	M2	1996	−52.807°	77.546°	Geolocator
Adult	Male	M5	1996	−51.242°	177.309°	Geolocator
Adult	Male	72446	2007	−41.852°	106.172°	Satellite telemetry
Adult	Unknown	U4	1996	−46.727°	181.569°	Geolocator
Juv	Female	1392	2001	−38.123°	45.196°	Satellite telemetry
Juv	Female	8959a	2001	−39.996°	62.114°	Satellite telemetry
Juv	Female	9058	2001	−39.654°	49.045°	Satellite telemetry
Juv	Female	11817	2001	−40.055°	78.897°	Satellite telemetry
Juv	Female	25751	2002	−37.710°	44.748°	Satellite telemetry
Juv	Female	38558	2002	−37.294°	117.143°	Satellite telemetry
Juv	Female	38559	2002	−39.015°	57.069°	Satellite telemetry
Juv	Male	1391	2001	−38.337°	89.743°	Satellite telemetry
Juv	Male	8959b	2002	−38.200°	87.430°	Satellite telemetry
Juv	Male	8960a	2001	−40.489°	68.226°	Satellite telemetry
Juv	Male	8960b	2002	−42.275°	128.003°	Satellite telemetry
Juv	Male	9059	2001	−39.942°	70.927°	Satellite telemetry
Juv	Male	38557	2002	−41.938°	132.455°	Satellite telemetry

### Initial migration and direction of departure flights

**Figure 1:**
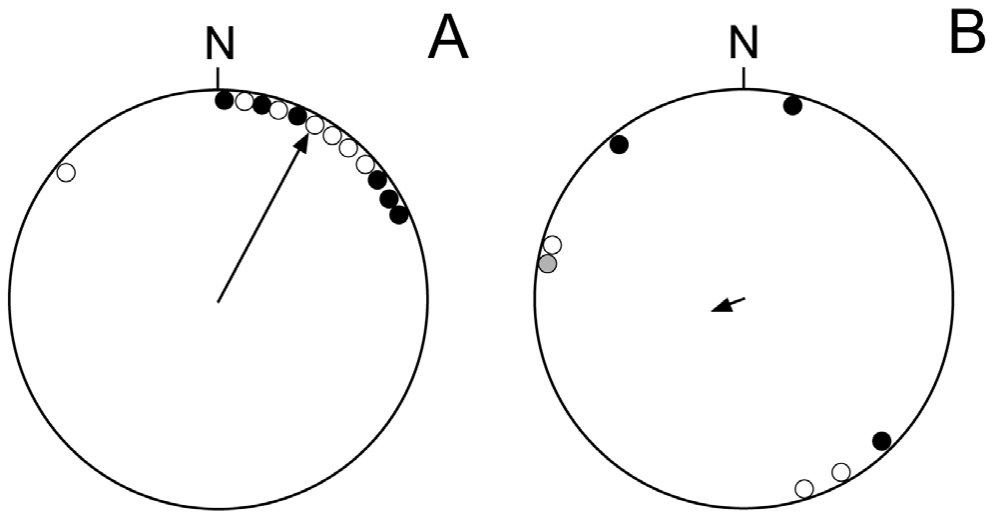
Mean orientation of initial departure flights of wandering albatrosses departing from Crozet Islands. (A) Departure flights by juvenile wandering albatrosses. (B) Departure flights by adult wandering albatrosses. The calculations are based on the initial flight segments covering on average 600 km from the breeding sites at Crozet Islands. The birds were tracked by satellite telemetry (13 juveniles, 2 adults) or light loggers (5 adults). The departure directions for females are indicated by open circles, while filled circles denote departures by males. Grey circle denotes an adult with unknown sex. For further information see methods section.

**Figure 2:**
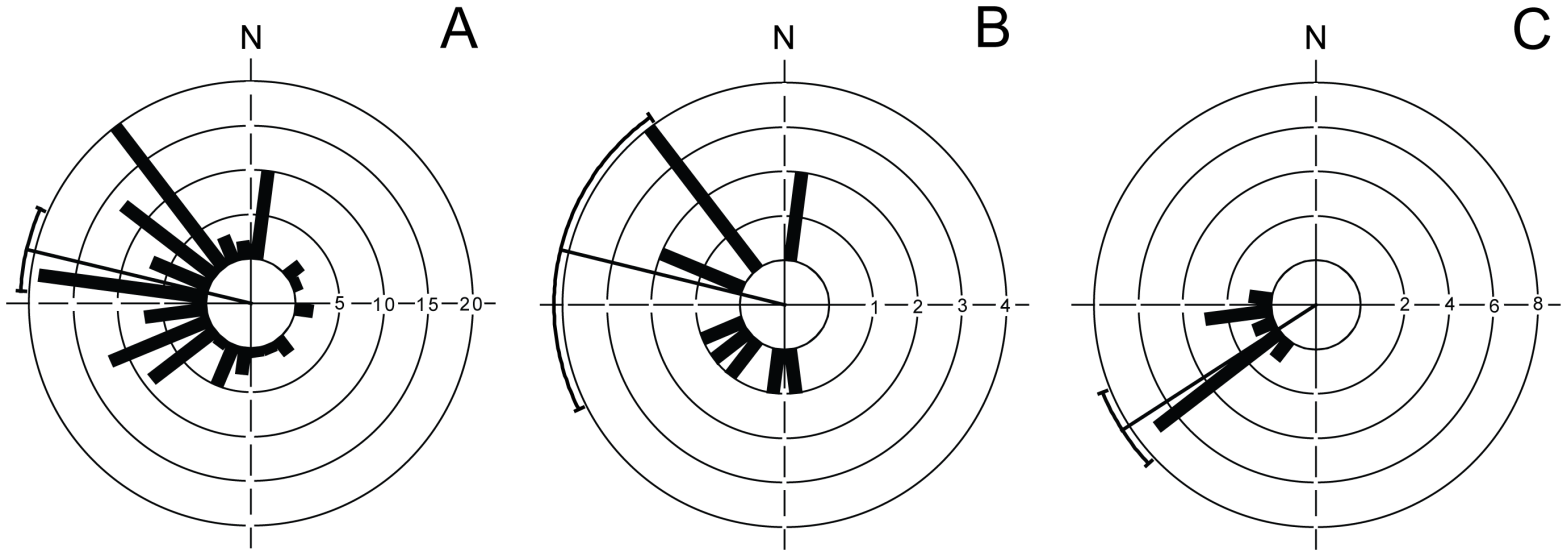
Average wind directions based on estimated winds per day at Crozet Island. (A) Average wind direction in November and December in 2001 and 2002. (B) Average wind direction the day before juvenile wandering albatrosses with transmitters initiated their prolonged flights towards north-east, (C) Average wind direction at day of departure. Average daily winds at Crozet Islands were extracted from QuickSCAT. Mean wind direction and 95% confidence intervals are given.

**Figure 3:**
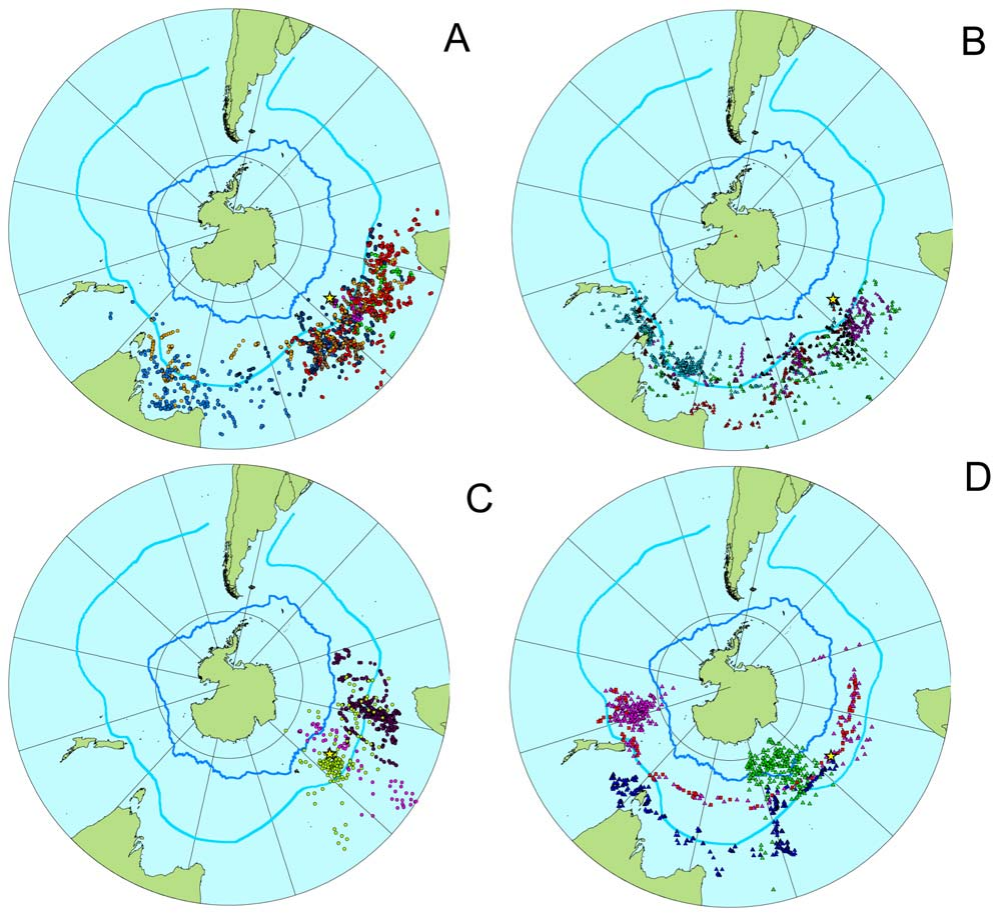
Satellite track positions of individual female and male wandering albatrosses of different age classes migrating from Crozet Islands in November and December 2001–2007. (A) Juvenile females. (B) Juvenile males. (C) Adult females. (D) Adult males. The starting point located at the Crozet Island is indicated by a star and the Sub-Tropical (to the north) and Sub-Antarctic (to the south) Fronts are indicated by blue lines. Individual tracks of female wandering albatrosses are indicated by coloured circles, while the tracks by males are indicated by coloured triangles. The migration route by an adult bird of unknown sex (D) is indicated by squares. The map is a gnomonic polar projection.

All departing juvenile wandering albatrosses showed very similar migration behaviour, during the first weeks after they left the breeding island without any assistance from their parents. Typically the juveniles departed from the breeding island by a short flight and thereafter immediately landed on water. At this stage they were sitting mostly on the water, only drifting slowly with the sea currents during the first 1–15 days off the island. The juveniles apparently waited for days with wind assistance from south-west to initiate rapid directional flights to the north to north-east (Figs. 1, 2, 3), with a mean direction significantly different from random (α = 28°, r = 0.89, n = 13, p<0.001, Rayleigh test, Fig. 1a). Only one individual was selecting a prolonged and straight flight directed towards north-west (Fig. 1a). This female juvenile later ended up in a foraging zone to the north-west of the Crozet Islands (red circles in Fig. 3a). There was no difference in initial mean departure directions between female (α = 20°, r = 0.87, n = 7, p<0.01, Rayleigh test) and male (α = 36°, r = 0.92, n = 6, p<0.01, Rayleigh test) young albatrosses (U^2^ = 0.08, df = 6, p>0.05, Watson's U^2^-test). The section of prolonged directed flight tracks by the juvenile wandering albatrosses lasted 1–4 days and covered mean distances of 600 km (range: 260–1010 km) until they crossed the subtropical convergence and initiated meandering flight paths in subtropical waters, ending in a restricted area of the ocean (Fig. 3).

The adult wandering albatrosses left the breeding colonies at Crozet Islands for a sabbatical year at sea in directions not significantly different from random (α = 248.3°, r = 0.13, n = 7, p>0.05; Fig. 1b). The orientation of the departure flights differed significantly between juvenile and adult wandering albatrosses (U^2^ = 0.24, df = 7, p<0.02, Watson's U^2^-test), demonstrating different preferred departure directions between age groups (Fig. 1).

The daily wind direction at Crozet Islands were on average north-west to west in November and December in 2001 and 2002, when young wandering albatrosses normally leave the breeding island (α = 285.1°, r = 0.62, n = 118, p<0.001, Fig. 2a). The juvenile albatrosses selectively departed with south-westerly winds (α = 237.1°, r = 0.96, n = 13, p<0.001, Fig. 2c), i.e. winds blowing towards north-east. There was a significant difference in average wind direction for the day of departure compared to the average wind direction estimated for the day before departure (α = 293.6°, r = 0.55, n = 13, p<0.05, Fig. 2b) (U^2^ = 0.34, df = 13, p<0.01, Watson's U^2^-test), suggesting the juvenile albatrosses were waiting for suitable winds and actively selecting days of departure for their long and directed initial flights when they experienced tailwinds towards north-east. All juvenile birds selected very similar wind conditions for departure, even though their individual departures occurred on different days from 25 November to 13 December in 2001 and 27 November to 10 December in 2002.

### Flight departure relative to destination area at sea

After the initial departures following relatively straight paths to the north to north-east, the young albatrosses started more circuitous movements in the sub-tropical waters moving towards specific zones of the Southern Ocean (Fig. 3). The flight paths within the foraging zones where they remained for a longer time were characterized by shorter flights in various directions during which time they sometimes returned to a region of the ocean where they had already been and crossed previous paths of the route. These meandering flights were initiated by most females when crossing the Sub-tropical Convergence zone, while males were heading for more distant foraging zones and continued their prolonged segments of more straight flight paths to the east before the circuitous flights within a restricted ocean zone were initiated (Fig. 3).

**Figure 4:**
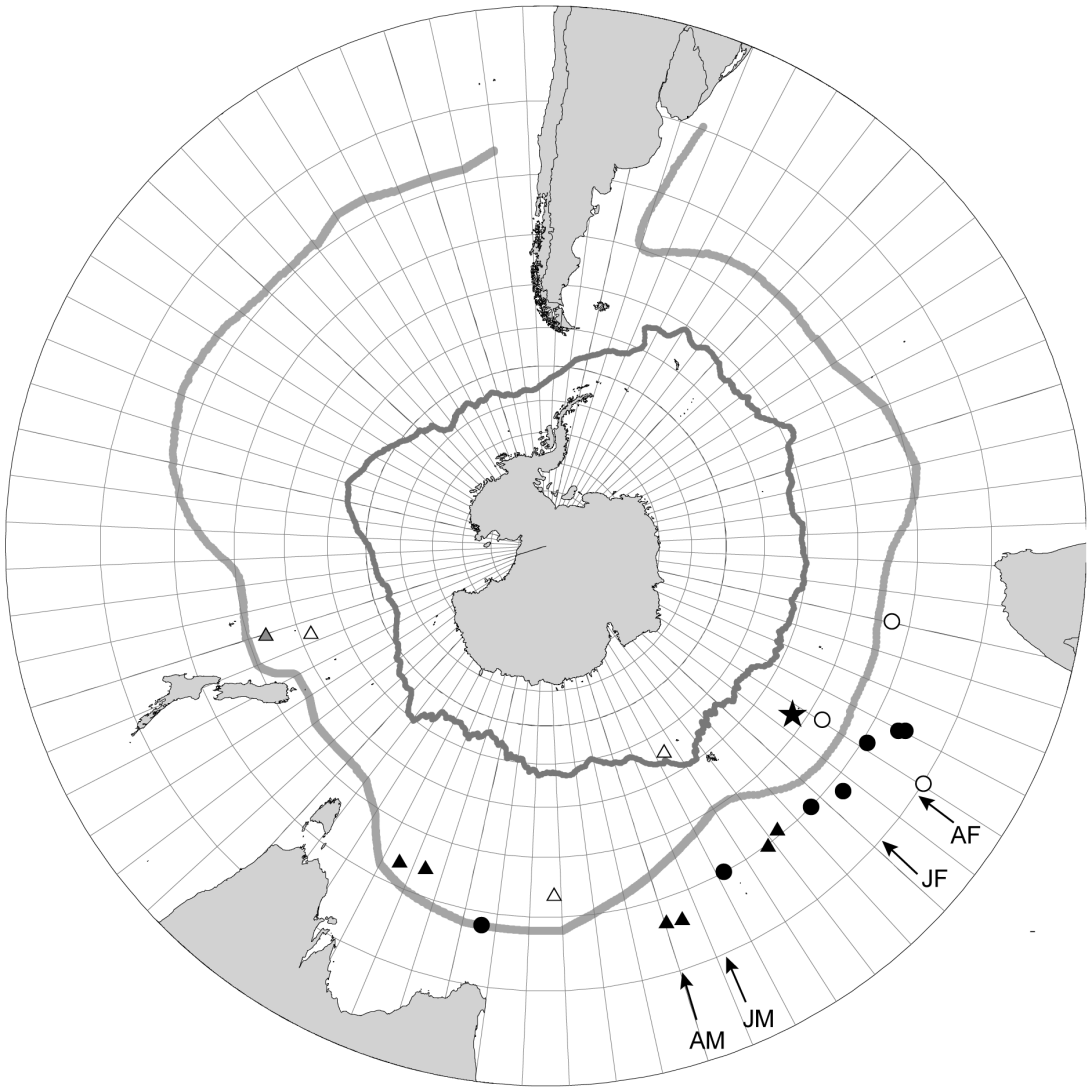
Locations of the centre of preferred ocean foraging zones for individual wandering albatrosses. Positions refer to different age and sex classes tracked by satellite telemetry during the migration from the breeding sites at Crozet Islands. Filled circles: juvenile females, open circles: adult females, filled triangles: juvenile males, open triangles: adult males. The grey triangle refers to an adult of unknown sex. Mean longitudes for each age category (JF: juvenile females, AF: adult females, JM: juvenile males, AM: adult males) are indicated by arrows. The starting point located at the Crozet Island is indicated by a star and the Sub-Tropical and Sub-Antarctic Fronts located around the Antarctic continent are indicated by light grey and dark grey lines, respectively.

There was a clear difference between sexes for both age classes in the ocean destination area (Fig. 4), in which the females (mean longitude = 58.3°E, S.E. = 10.3) stayed in areas closer to the breeding sites at Crozet Islands and the males (mean long. = 108.2°E, S.E. = 10.6) headed for areas more to the east (GLMM: F_3,18_ = 4.51, P<0.05; Fig. 4, Table 1). The juveniles headed for ocean areas of similar longitude sections that was used by non-breeding adult albatrosses (adult females: mean long. = 43.1°E, S∶E: = 6.6 ; adult males: mean long. = 120.3°E, S.E. = 29.6). Males on average migrated to areas further away from the breeding island (mean GC distance as calculated from Crozet Island to the centre of the foraging areas = 3579 km, S.E. = 779) compared to females (mean GC distance = 1460 km, S.E. = 421) (t-test, t = −2.78, df = 18, p<0.05). Within all four years with tracking data the males moved further to the east than females, and which was true both for adult and juvenile wandering albatrosses.

**Figure 5:**
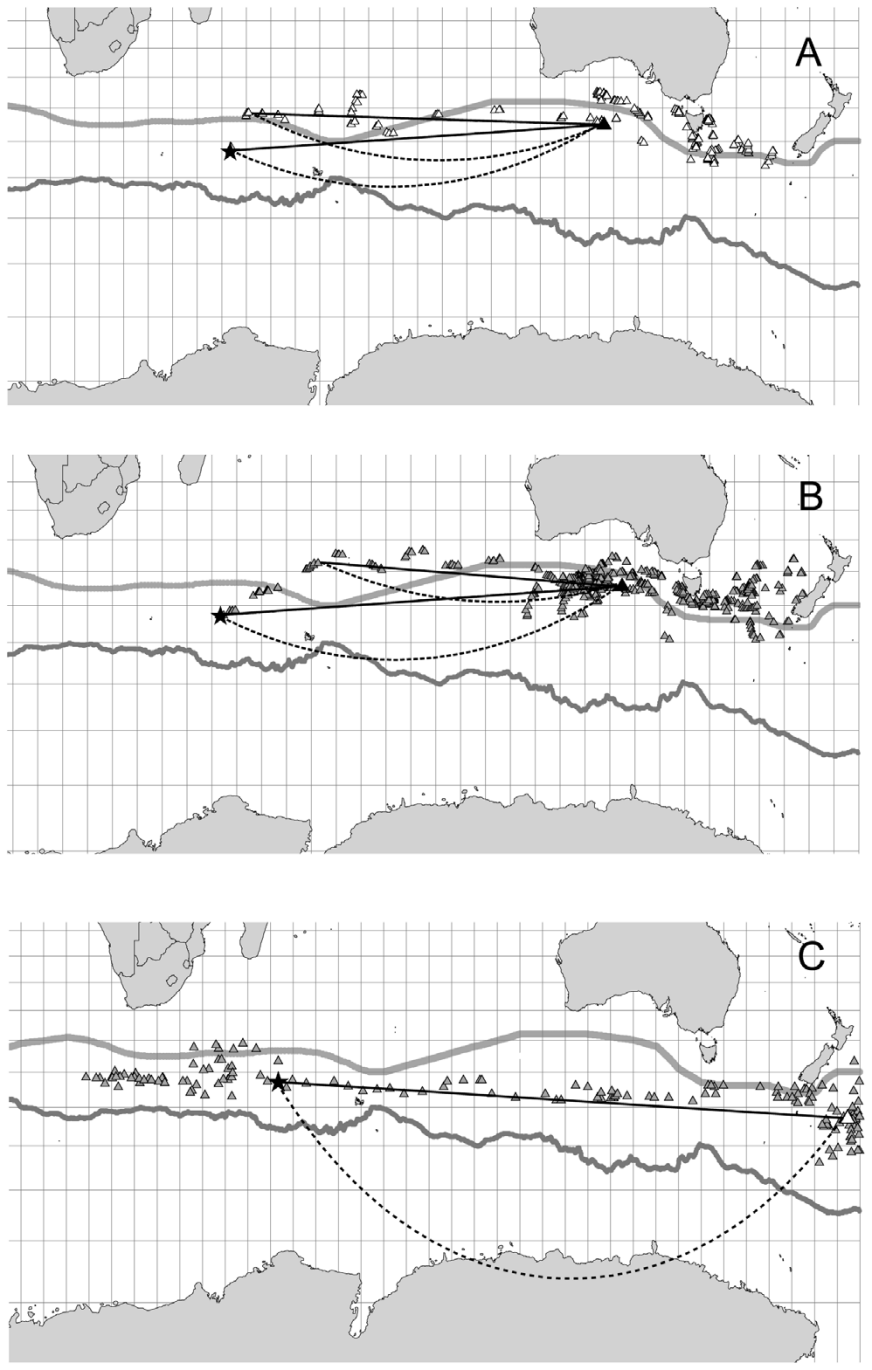
Examples of flight routes of two young male (A, B) and an adult male wandering albatross departing from breeding locations at Crozet Islands as recorded by satellite telemetry. Indicated are great circle (filled line) and rhumbline (broken line) routes from the breeding sites at Crozet Islands and from a location where the individual albatross had covered the initial departure flight and initiated prolonged fast movements to the destination areas. The location of the Crozet Islands is indicated by a star. The map is a Mercator projection.

We found a difference in latitude of the preferred foraging zones between sexes (all females: mean latitude −39.4°S, S.E. = 1.02; all males: mean latitude = −43.0°S, S.E. = 1.04) (GLMM: F_3,18_ = 7.88, P<0.01), but also between age groups in which on average adults preferred areas more to the south of the juvenile birds (adults: mean latitude = −44.4°S, S.E. = 1.2; juveniles: mean latitude = −39.6°S, S.E. = 0.8). However, the adult females preferred zones more to the north compared to adult males (adult females: mean latitude = −40.0°S, S.E. = 2.6; adult males: mean latitude = −48.6°S, S.E. = 3.4; Figs. 3 and 4). The 75% kernel density zones showed migration to ocean areas located further east in male adult wandering albatrosses compared to adult females (Fig. 5), and more overlapping zones for juvenile birds. However, when the initial period was separated from the second part of the tracking period, the 75% kernel density zones for juvenile male and female wandering albatrosses showed a similar difference in preferred foraging zones as in adults. These results show that the juvenile males gradually move to more distant areas to the east, but at a slightly later time relative to the adult males while juvenile females reached the preferred foraging zones earlier than the males.

The juvenile wandering albatrosses were following routes from the breeding island to the preferred oceanic foraging zone differing from both a direct rhumbline (i.e. constant geographic course) and a great circle (i.e. shortest distance) route (Fig. 5). The juveniles showed very distinct north to north-easterly routes initially, but thereafter changed to either predominantly westerly (females) or easterly (males; Fig. 5A) flight routes. For females the meandering foraging flights started earlier than for males. We therefore reject both hypothesis 1, and 2, but consider hypothesis 3 representing a combination of routes to explain the initial dispersal migration of juvenile wandering albatrosses. The adult wandering albatrosses engaging in long transport flights to distant foraging zones, on the other hand, seemed to follow approximately rhumbline routes to their destinations starting at the breeding island along more southerly latitudes than the juvenile birds migrating to the same geographical region (Fig. 5). The juvenile birds all initiated their departures with flights directed mainly northeast, and thereafter the males engaged in more eastern movements, while the females at departure headed towards foraging zones to the north and northwest of Crozet Islands. We thus, reject also hypothesis 4, predicting that the juveniles followed similar routes as adult birds when departing from the same breeding colony.

**Figure 6:**
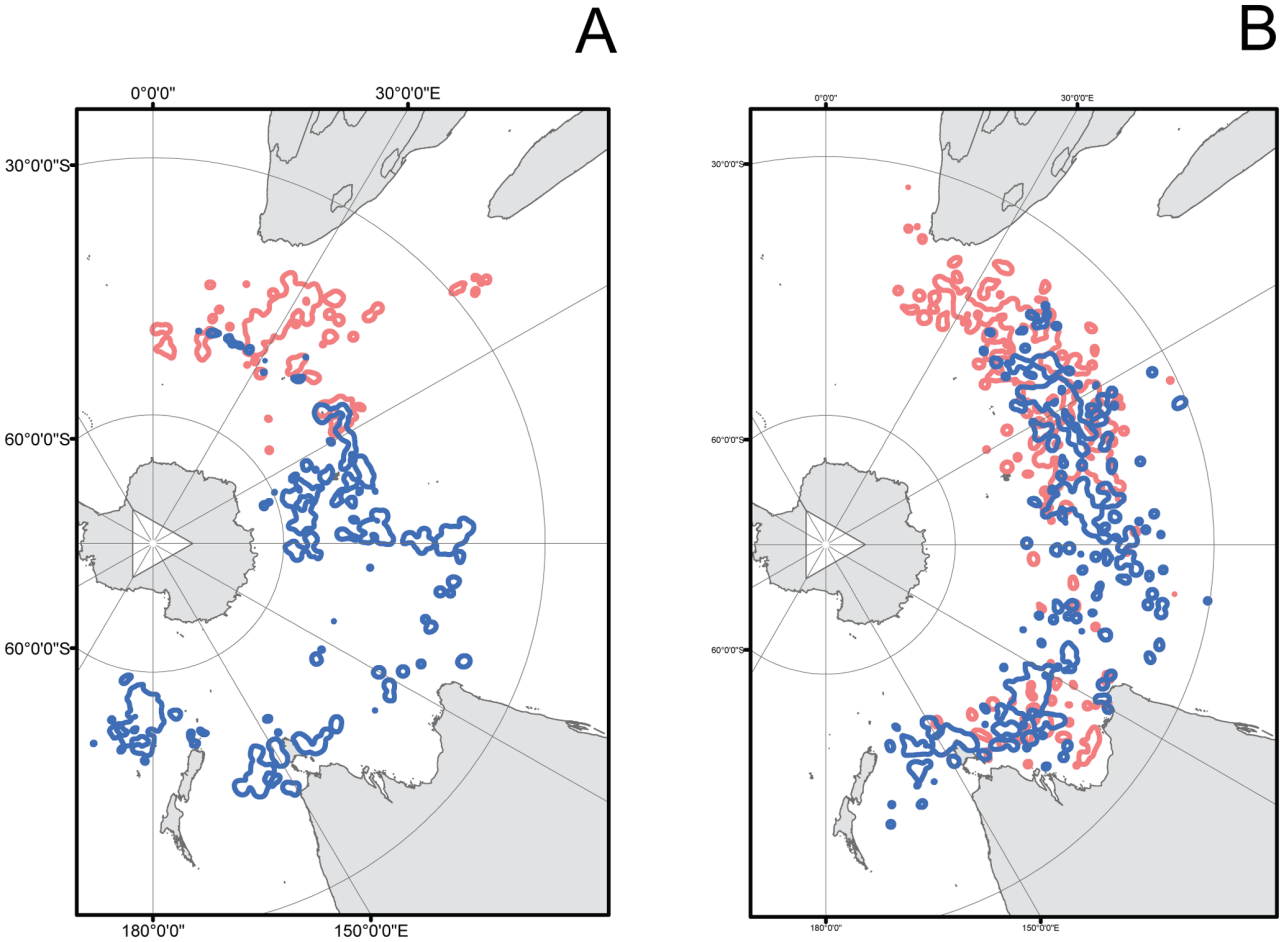
Post-breeding foraging zones explored by juvenile and adult wandering albatrosses breeding at Crozet Islands during the post-breeding period as indicated by 75% kernel density zones. (A) Adult females (red) and males (blue). (B) Juvenile females (red) and males (blue). The location of Crozet Islands is indicated by a star.

**Figure 7:**
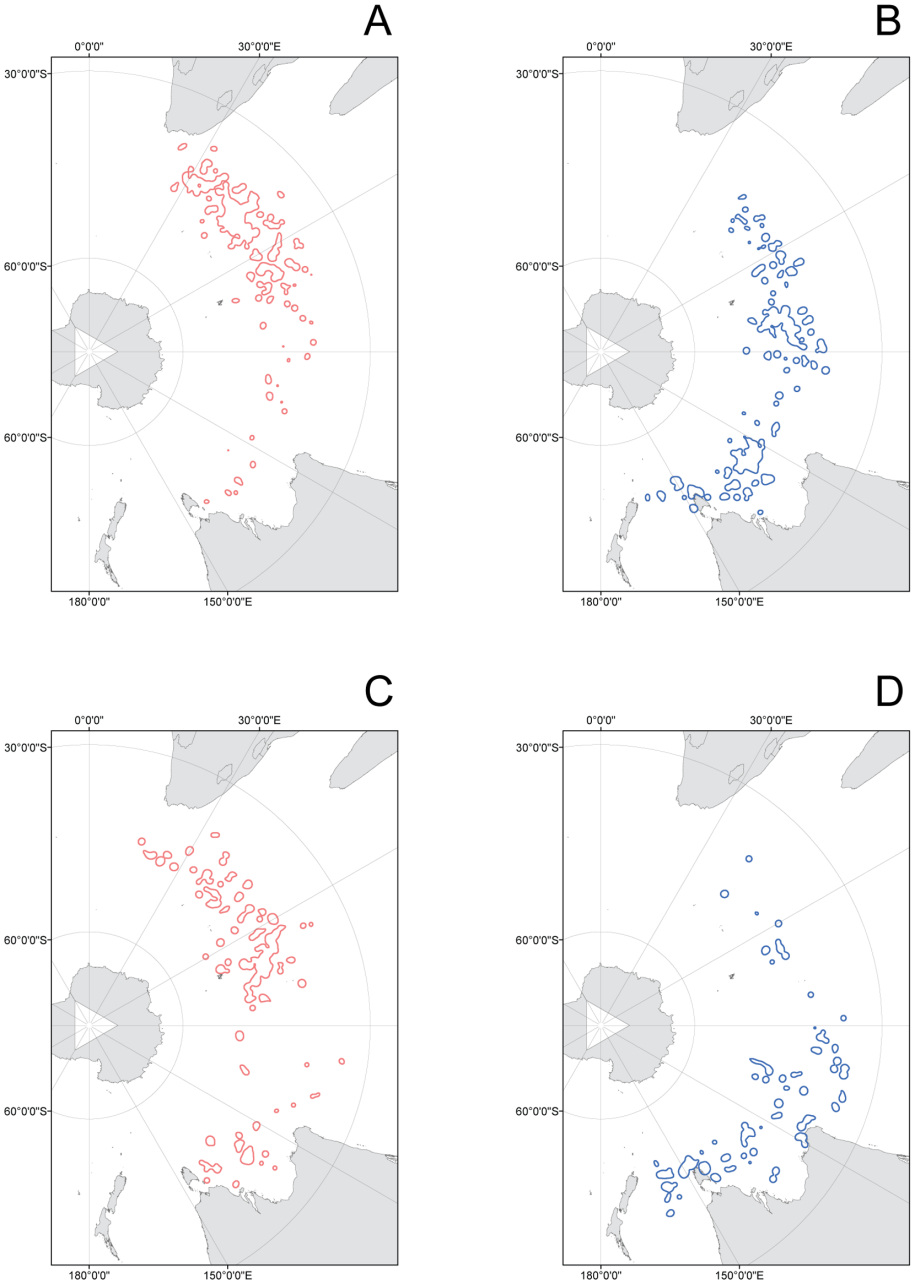
Ocean foraging zones for female and male juvenile wandering albatrosses migrating from Crozet Island as indicated by 75% kernel density zones for different time periods. (A) Juvenile females during January and February. (B) Juvenile males during January and February. (C) Juvenile females during March and April. (D) Juvenile males during March and April. The location of Crozet Islands is indicated by a star.

The plots of the 75% kernel density isolines showed that adult Crozet Island wandering albatrosses had very limited overlap between the sexes in the ocean distributions (Fig. 6). The 75% kernel density zones for the juvenile female wandering albatrosses further showed that they migrated to the preferred ocean areas in the southern part of the Indian Ocean shortly after they left the breeding island, and then remained in the same area during most of the remaining tracking period (Fig. 7 A and C). Juvenile male wandering albatrosses on the other hand moved gradually eastwards from Crozet Island and reached the destination areas in March to April, after the two initial tracking months had passed (Fig. 7 B and D).

## Discussion

The satellite tracks of juvenile wandering albatrosses dispersing from Crozet Islands revealed that, during their first migration, they follow a sequence of inherited behavioural steps according to which they are guided to reach their individually preferred oceanic foraging destinations, coinciding with the adults preferred and sex-segregated ocean areas and thus probably used also later in life [3], [4], . We could identify a typical sequential pattern of behaviours in the departure migration flights by which the juvenile albatrosses: 1) departed from the nest site and landed on water, 2) sitting mainly on the water they were drifting with the currents, possibly engaging in short flights and foraging during which time they waited for suitable winds assisting them to fly north of the island, 3) they initiated north to north-easterly flights with tailwind assistance from south-west and continuing their flights for on average 600 km crossing the Sub-tropical Convergence Zone, 4) they initiated meandering foraging flights in an oceanic region where they remained for the rest of the tracking period lasting several months (young females), or continued on prolonged transport flights to the east along a certain latitude section, sometimes remaining in an ocean area for a few weeks, and then continuing further east to the ocean basin south from Australia and New Zealand where they remained for the rest of the tracking period (young males).

Feeding are expected to occur continuously during the dispersal movements, and predominantly at daytime as wandering albatrosses search for scarce prey at the surface such as dead squids and fish, but with higher success at polar fronts and upwelling zones [22]. The observed sequence of behavioural steps are performed by young wandering albatrosses adapted to an open ocean environment, in which they are largely affected by winds for their movements, either assisting them to reach destination areas or sometimes causing them to be drifting away from their intended goal. Most likely the genetic migratory program and its specific behavioural components have evolved under strong influence from the winds for the wandering albatrosses, as the winds in the Southern Ocean show predominant and reliable westerly to north-westerly directions over the year. The predominantly westerly winds are extending over a large latitude section of the ocean, but easterly winds are found further south along the Antarctic continent, as well as in the northern limit of the species range [23], [24].

Our results show that the juvenile wandering albatrosses selectively use winds during their departure flights from the breeding island, and that all juveniles selected to depart in winds that would take them to the north-east before they initiated their extended flights away from the ocean near the Crozet Islands. Adult breeding wandering albatrosses from Crozet Islands have been shown to fly with tail- or side-winds during their foraging flights, resulting in stereotypical flight patterns following large loops [4], [31]. Predictable weather systems are thought to be used to exploit tailwinds during these flights, and thus, to save energy [23]. However this behaviour is for central place foraging breeding birds that have to return to the colony at each trip: the optimal use of wind is crucial for the birds to be able to return without having head winds [23]. Although the constraints are not the same, the juvenile wandering albatrosses seem to use a similar strategy to favour tailwinds during their initial flights to the north-east, possibly reacting to other parameters than wind, e.g. air pressure, to predict the approaching low pressure systems and tailwind conditions. Pigeons *Columba livia* have been shown to be sensitive to air pressure changes [25], and the departure of migratory birds have frequently been shown to be correlated with changes in air pressure and related weather parameters associated with favourable weather and tailwind conditions for migration flights, e.g. [26]–[30]. Our results suggest a similar behavioural adaptation to predict and react to winds in a selective way in departing juvenile wandering albatrosses.

Our satellite tracks show the ability of juvenile wandering albatrosses to follow as expected meandering flights which may lead to revisits of sections of ocean they already passed [4], but also to remain within specific latitude sections, but also engagement in longer more directed flights during migration. Both these behaviours suggest the use of an inherited migration, or dispersal, program with navigation ability in young albatrosses, rather than use of a strict clock-and-compass orientation [12], [13], where in its strictest form and applied to migrating songbirds course corrections are not considered to be included [31]. Furthermore, some of the juvenile female wandering albatrosses heading for wintering areas north-west of the breeding island have to fly opposite to the dominating wind direction to reach these sectors of the ocean, cf. [23]. This migration pattern is not easily explained only by a simple clock-and-compass model and selective use of tailwinds taking the birds in a certain direction for extended periods of time, but rather requires more advanced and selective use of winds different from that required during the initial part of migration and, for example, leading to directions of movements in the opposite directions of males for these females. Our data suggest that rather than showing a population-specific migration destination typical for many migratory songbirds, the migration program of juvenile wandering albatrosses from Crozet Islands seems to rely on a set of behavioural steps, i.e. similar but sex-specific migration behaviours, which will lead them different distances across the sea and to sex-specific ocean areas already during their first migration. In addition to this migration phenotype, navigation skills are needed to enable the albatrosses to correct for wind drift and return to productive and already visited foraging zones of the ocean. The sensory basis of such navigation program used by the albatrosses is not known [26], but needs to be investigated.

### Flight routes between ocean areas

The flight routes taken by juvenile wandering albatrosses from the breeding island to the oceanic foraging zone used later in the year did not follow closely great circle (ortodrome) or rhumbline (loxodrome) routes (Fig. 5). Rather their transport flight routes following general latitude sections are influenced by winds leading to large variations in track directions between nearby locations, and thus leading to routes much longer than the expected great circle and rhumbline routes, probably because they forage during these flights. A combination of sections of different preferred routes seems therefore best to explain the young wandering albatrosses initial dispersal migration flights, i.e. first north-east and thereafter predominantly east for juvenile males, and north to north-east and thereafter predominantly westerly flights in young females. Furthermore, the route following must be considered as approximations, given the small and medium scale circuitous flight paths relative to winds that is typical for wandering albatrosses using dynamic soaring and limitations in positions received per day from satellite tracking technology used in this study [32], [33].

How would the albatrosses keep their courses during ocean movements? A rhumbline route corresponds to a constant geographic course, and could be kept by a stellar compass which is dependent on the rotation centre of the sky, such that the selected course will be kept irrespective of the longitudinal movement, e.g. [11], [15], [34]. A sun compass can in theory be used, but is not unaffected by crossings of the longitudes. The birds will have to reset their internal daily clock with the local time in order to use a sun compass, as course changes will otherwise accumulate when the bird move across longitudes [34]. The rhumbline route is slightly longer than the great circle route, for which the latter corresponds to the shortest route between two locations on the globe, e.g. [35], [36]. In order to save energy during transportation, following great circles would in many cases be most beneficial [2], since the distance between two sites is minimized. In the case of the wandering albatross, moving between distant locations across the Southern Ocean flying along long stretches of the Antarctic Continent a great circle route is not applicable. Following such a route would take the bird away from a suitable foraging zone to areas more to the south with more ice, colder temperatures and especially headwinds when moving east at the margin of the Antarctic Continent (example of track given in Fig. 5b; cf. [23]). Remaining within the latitudinal sector of the Southern Ocean around −40°S and south thereof, with strong and predominantly north-westerly to westerly winds, would be the best strategy in terms of foraging efficiency and transportation cost per unit distance for the wandering albatrosses moving to distant locations, rather than minimizing the distance between two locations by flying along approximate great circles. The young male wandering albatrosses tracked in our study seem to follow this flight zone (−40°S and S thereof) and explore the winds in order to gain tailwinds during transportation.

### Sex-specific migration to different ocean areas

Our satellite data show that female and male wandering albatrosses from Crozet Islands migrate to different geographical regions, where females move to areas of the South Indian Ocean and males predominantly migrate to foraging zones further to the east of the Indian Ocean, south from Australia and the Tasman Sea, and up to the eastern Pacific Ocean. An important result of this study is the preferences to migrate to the sex-specific ocean areas present already in juvenile albatrosses migrating for the first time, suggesting this difference in migration performance is part of the inherited migration program and expressed during independent migrations without guidance from parents. In contrast to many other bird species where males migrate the shortest distances from the breeding sites [37], the female wandering albatrosses on average end up in ocean areas closer to the breeding colonies compared to the males. In passerine migrants, several evolutionary explanations to differential migration have been suggested, related to e.g. foraging competition, body size, dominance, and time of arrival [37]. Female wandering albatrosses are smaller than males [38], and thus, their more northerly ocean distributions compared with males [39] was supposed to be a result of foraging competition in which subdominant females are progressively forced to select ocean areas of lower quality, e.g. [23], [37]. However, our results show that outside the breeding season males and females differ also in the longitudinal distribution, males moving farther from the colonies, and that this difference is not set progressively as a result of competition, but is inherited.

During the breeding season, adult wandering albatrosses are central place foragers and have to return frequently to the nesting sites: at this time, females are also foraging over more northerly waters than males [39]. In fact the breeding and non-breeding foraging zones explored by female wandering albatrosses at Crozet Islands largely overlap [39], suggesting that shorter dispersal movements are favoured by year around availability of food in this sector of the ocean. The females' food preference and physiological and morphological adaptations to higher temperatures and lower wind speeds could, thus, be a proximate explanation to the differential migration and sex-segregated foraging zones located more to the north in adult females. However, it is likely that the genetically encoded behaviour in juveniles guiding the different sexes to different oceanic foraging areas has been selected to avoid competition in the past, e.g. [40]. This is also suggested by the strict foraging of young birds in waters that are much less productive than those exploited by adult birds [21]. Most likely the inherited migration program leading to differential migration in wandering albatrosses is a result of the combined selective pressures from foraging competition and necessary physiological adaptations, such as longer wings in juveniles enabling them to forage in areas with lower wind speeds than the adults [38], but also showing the general aerodynamic adaptations to the extreme and windy environment of the Southern Ocean. The wandering albatross seems to be an excellent example of an organism, where the evolution of long-distance movements across the globe is a result of interactions between the birds' endogenous migration program, its motion and navigation capacity, foraging competition and physical factors of the external environment. In the wandering albatross we believe these interactions and associated selective forces have led to the evolution of sex-segregated foraging zones used during breeding, but also during the non-breeding period expressed already during the first migration in juvenile wandering albatrosses.

## Materials and Methods

### Animals and study site

We used satellite telemetry and light loggers (i.e. geolocators, British Antarctic Survey, UK) to record the wandering albatross migration to foraging areas at sea. We attached satellite transmitters (Microwave PTT 100, with batteries or solar panels) to 13 (6 males and 7 females) fully feathered juvenile birds in mid November 2001 (7 individuals) and 2002 (6) at the Crozet Islands, south-western Indian Ocean [21]. The six solar panel powered 35–50 g satellite transmitters we used were programmed with a duty cycle of 10 h on and 24 h off. We also used seven 35–45 g battery powered satellite transmitters with a duty cycle of 10–18 h on and 54 h off. All satellite transmitters were fitted with adhesive tape on the back feathers of the birds, and fell off when the birds moulted. The locations of transmitters were determined using the Argos system (http://www.argosinc.com/), and were normally resulting in between 4 and 14 uplinks per day. We filtered all locations obtained (all classes) following Weimerskirch et al. [39] by which locations which necessitated a speed of travel of >90 km/h were removed, as well as locations that were obtained at less than 10 minute intervals.

The movements of juvenile wandering albatrosses were compared with those recorded by light intensity loggers for adult birds (five individuals) breeding at the same Island as the juveniles and tracked during the non-breeding period [3]. We also included in our analyses tracks of two adult wandering albatrosses using 45 g solar panel GPS satellite transmitters (Microwave 100, solar GPS) with a duty cycle of 10 h on and 24 h off. These adults were fitted with satellite transmitters before leaving the island after a failed breeding attempt in 2007. In total seven adult wandering albatrosses were tracked (3 females, 3 males and 1 of unknown sex). Ethical permission to track wandering albatrosses by satellite telemetry and geolocators at Crozet Island was received from the Ethic Committee of the French Polar Institute (IPEV, Program n° 109) to Henri Weimerskirch.

### Data evaluation and statistics

Daily average wind directions at Crozet Islands were extracted from QuickSCAT (http://manati.orbit.nesdis.noaa.gov/doc/oppt.html). The position of fronts was taken from Belkin and Gordon [41]. The routes recorded by satellite telemetry for individual birds were compared with expected constant geographic courses, corresponding to straight lines on a Mercator map projection, and great circle routes corresponding to straight lines on a gnomonic map projection, e.g. [20], [36]. The shortest route between two locations on the globe corresponds to a great circle [35], along which the geographic course needs to be gradually shifted. Flying along a constant geographic course (rhumbline) will be slightly longer compared to a great circle route, e.g. [11], [34], [36].

Mean track directions for individual birds were used to calculate sample mean vectors (α) and axes of orientation according to standard procedures given in Batschelet [42]. Mean vector lengths (r), ranging between 0 and 1, give a measure of scatter that is inversely related to the angular scatter. The Rayleigh test was applied to test if a circular distribution differed significantly from random [42]. We used the Watsons's U^2^-test to analyze differences in orientation between groups [42]. All circular statistics was performed using Oriana 2.1 and by consulting Batschelet [42]. We used general linear mixed models (GLMM) to assess the effect of sex and age as categorical variables on the latitude and longitude distributions of the centre of the foraging zones selected by individual birds. All GLMM analyses were performed with JMP (Statistical Discovery 2005, © SAS Institute). We used the fixed kernel method [43] with least squares algorithm [44] and a smoothing factor of 1° to calculate contours including 75% of the locations [45] for adults and for juvenile albatrosses breeding at Crozet Islands. The kernel density contour maps were produced by ArcGIS 10.

## References

[1] Jouventin P, Weimerskirch H (1990) Satellite tracking of wandering albatrosses. Nature 343: 746–748.

[2] Weimerskirch H, Doncaster P, Cuénot-Chaillet F (1994) Pelagic seabirds and the marine environment: foraging patterns of wandering albatrosses in relation to prey availability and distribution. Proc R Soc Lond B 255: 91–97.

[3] Weimerskirch H, Wilson RP (2000) Oceanic respite for wandering albatrosses. Nature 406: 955–956. 10984040 10.1038/35023068

[4] Åkesson S, Weimerskirch H (2005) Albatross long-distance navigation: comparing adults and juveniles. J Nav 58: 365–373.

[5] Warham J (1990) The petrels: their ecology and breeding systems. London: Academic Press.

[6] Papi F, Luschi P (1996) Pinpointing ‘Isla Meta’: the case of sea turtles and albatrosses. J Exp Biol 199: 65–71. 9317352 10.1242/jeb.199.1.65

[7] Åkesson S (1996) Geomagnetic map used for long-distance navigation? Trends Ecol Evol 11: 398–400. 21237895 10.1016/0169-5347(96)30040-2

[8] Åkesson S (2003) Avian long-distance navigation: experiments with migratory birds. In: Berthold P, Gwinner E, editors. Bird Migration. Berlin, Heidelberg: Springer-Verlag. pp. 471–492.

[9] Åkesson S, Alerstam T (1998) Oceanic navigation: are there any feasible geomagnetic bi-coordinate combinations for albatrosses? J Avian Biol 29: 618–625.

[10] Bonadonna F, Benhamou S, Jouventin P (2003) Orientation in “featureless” environments: the extreme case of pelagic birds. In: Berthold P, Gwinner, E, editors. Bird Migration. Berlin, Heidelberg: Springer-Verlag. pp. 367–377.

[11] Åkesson S, Hedenström A (2007) How migrants get there: migratory performance and orientation. BioScience 57: 123–133.

[12] Gwinner E (1986) Circannual Rhythms. Endogenous Annual Clocks in the Organization of seasonal Processes. Berlin, Heidelberg: Springer-Verlag.

[13] Berthold P (1996) Control of Bird Migration. London: Chapman & Hall.

[14] Able KP (1980) Mechanisms of orientation, navigation and homing. In: Gauthreaux S, editor. Animal Migration, Orientation and Navigation. New York: Academic Press, pp. 283–373.

[15] Emlen ST (1975) Migration: orientation and navigation. In: Farner DS, King JR, editors. Avian Biology. Vol. 5. New York: Academic Press. pp. 129–219.

[16] Wiltschko R, Wiltschko W (1995) Magnetic Orientation in Animals. Berlin, Heidelberg: Springer-Verlag.

[17] Rabøl J (1978) One-direction orientation versus goal area navigation in migratory birds. Oikos 30: 216–223.

[18] Fransson T, Jakobsson S, Kullberg S (2005) Non-random distribution of ring recoveries from trans-Saharan migrants indicates species-specific stopover areas. J Avian Biol 36: 6–11.

[19] Fransson T, Jakobsson S, Johansson P, Kullberg C, Lind J, et al (2001) Magnetic cues triggers extensive refuelling. Nature 414: 35–36. 11689932 10.1038/35102115

[20] Snyder JP (1993) Flattening the Earth. Two Thousand Years of Map Projections. Chicago and London: The University of Chicago Press.

[21] Weimerskirch H, Åkesson S, Pinaud D (2006) Postnatal dispersal of wandering albatrosses: implications for the conservation of the species. J Avian Biol 37: 23–28.

[22] Weimerskirch H, Doncatser CP, Cuenot-Chaillet F (1994) Pelagic seabirds and the marine environment: foraging patterns in wandering albatrosses in relation to prey availability and distribution. Proc R Soc Lond B 255: 91–97.

[23] Weimerskirch H, Guionnet T, Martin J, Shaffer SA, Costa DP (2000) Fast and fuel efficient? Optimal use of wind by flying albatrosses. Proc R Soc Lond B 267: 1869–1874. 10.1098/rspb.2000.1223 PMC1690761 11052538

[24] Weimerskirch H, Louzao M, de Grissac S, Delord K (2012) Changes in wind pattern alter albatross distribution and life-history trait. Science 335: 211–214. 22246774 10.1126/science.1210270

[25] Kreithen ML, Keeton WT (1974) Detection of changes in atmospheric pressure by the homing pigeon, *Columba livia* . J Comp Physiol B 89: 73–82.

[26] Åkesson S, Hedenström A (2000) Selective flight departure in passerine nocturnal migrants. Behav Ecol Sociobiol 47: 140–144.

[27] Åkesson S, Walinder G, Karlsson L, Ehnbom S (2001) Reed warbler orientation: initiation of nocturnal migratory flights in relation to visibility of celestial cues at dusk. Anim Behav 61: 181–189. 11170708 10.1006/anbe.2000.1562

[28] Åkesson S, Walinder G, Karlsson L, Ehnbom S (2002) Nocturnal migratory flight initiation in reed warblers: effect of wind on orientation and timing of migration. J Avian Biol 33: 349–357.

[29] Green M (2004) Flying with the wind – spring migration of Arctic-breeding waders and geese over south Sweden. Ardea 92: 145–160.

[30] Schaub M, Liechti F, Jenni L (2004) Departure of migrating European robins, *Erithacus rubecula*, from a stopover site in relation to wind and rain. Anim Behav 67: 229–237.

[31] Thorup K, Rabøl J (2001) The orientation system and migration pattern of long-distance migrants: conflict between model predictions and observed patterns. J Avian Biol 32: 111–119.

[32] Alerstam T, Gudmundsson GA, Larsson B (1993) Flight tracks and speeds of Antarctic and Atlantic seabirds: radar and optical measurements. Phil Trans R Soc Lond B 340: 55–67.

[33] Weimerskirch H, Bonadonna F, Bailleul F, Mabille G, Dell'Omo G, et al (2004) GPS Tracking of Foraging Albatrosses. Science 295: 1259. 10.1126/science.1068034 11847332

[34] Alerstam T (1996) The geographical scale factor in orientation of migrating birds. J Exp Biol 199: 9–19. 9317235 10.1242/jeb.199.1.9

[35] Imboden C, Imboden D (1972) Formel für Orthodrome und Loxodrome bei der Berechnung von Richtung und Distanz zwischen Beringungs-Wiederfundort. Vogelwarte 26: 336–346.

[36] Gudmundsson GA, Alerstam T (1998) Optimal map projections for analysing long-distance migration routes. J Avian Biol 29: 597–605.

[37] Ketterson ED, Nolan V Jr (1983) The evolution of differential migration. Curr Ornithol 1: 357–402.

[38] Shaffer SA, Weimerskirch H, Costa DP (2001) Functional significance of sexual dimorphism in wandering albatrosses, *Diomedea exulans* . Funct Ecol 15: 203–210.

[39] Weimerskirch H, Salamolard M, Sarrazin F, Jouventin P (1993) Foraging strategy of the wandering albatrosses through the breeding season: a study using satellite telemetry. Auk 110: 325–342.

[40] Clobert J, Danchin E, Dhondt AA, Nichols JD (2001) Dispersal. Oxford: Oxford University Press.

[41] Belkin IM, Gordon AL (1996) Southern ocean fronts from the Greenwich meridian. J Geophys Res 101: 3675–3696.

[42] Batschelet E (1981) Circular Statistics in Biology. New York: Academic Press.

[43] Worton BJ (1995) Using Monte Carlo simulation to evaluate kernel-based home range estimators. J Wildlife Manage 59: 794–800.

[44] Seaman DE, Powell RA (1996) An evaluation of the accuracy of kernel density estimators for home range analysis. Ecology 77: 2075–2085.

[45] Wood AG, Naef-Daenzer P, Prince PA, Croxall JP (2000) Quantifying habitat use in satellite-tracked pelagic seabirds: application of kernel estimation to albatross locations. J Avian Biol 31: 278–286.

